# Stability and adaptability of grain yield in quinoa genotypes in four locations of Iran

**DOI:** 10.3389/fpls.2024.1487106

**Published:** 2024-11-29

**Authors:** Vahid Jokarfard, Babak Rabiei, Ebrahim Souri Laki, Andreas Börner

**Affiliations:** ^1^ Department of Plant Production and Genetic Engineering, Faculty of Agricultural Sciences, University of Guilan, Rasht, Iran; ^2^ Department of Gene Bank, Institute of Plant Genetics and Crop Plant Research, Gatersleben, Germany

**Keywords:** grain yield, ideal genotype, mega-environment, genotype × environment interaction, multivariate methods

## Abstract

The genotype × environment interaction is one of the effective factors in identifying and introducing cultivars with stable grain yield in different environments. There are many statistical methods for estimating genotype × environment interaction, among which AMMI and GGE-biplot analyses provide better and more interpretable results. The objective of this study was to assess the genotype × environment interaction, as well as the adaptability and stability of 40 quinoa genotypes. The experiment was carried out in a randomized complete block design with three replications in eight environments (four locations of Iran and two years). The AMMI analysis of variance showed that the main effects of genotype and environment, as well as the interaction effect of genotype × environment were significant on grain yield. Separation of genotype × environment interaction based on the principal component method showed that the first six principal components were significant and accounted for 47.6%, 22.5%, 9%, 7%, 6% and 4.3% of the genotype × environment interaction variance, respectively. Based on the AMMI model, genotypes G16, G19, G35, G30, G39, G24, and G18 were identified as high-yielding and stable genotypes with high general adaptability. In contrast, genotypes G36, G27, G38, G9, G28, G29, G23, G34, G13, and G12 were the most unstable genotypes in the studied environments. In GGE-biplot analysis, two mega-environments were identified, and genotypes G16, G19, G25, and G17 were also identified as high-yielding and stable genotypes for these environments. Also, based on the biplot diagram of the ideal genotype, genotypes G16, G19, G17, and G35 were the nearest genotypes to the ideal genotype. In total, the results of various analyses showed that the three genotypes G16 and G19 were the superior genotypes of this experiment in terms of grain yield and stability. These genotypes can be introduced as high-yielding and stable genotypes to the climatic conditions of the studied areas.

## 1 Introduction

Quinoa (*Chenopodium quinoa* Willd.) is a crop plant from the *Amaranthaceae* family. The origin of this crop is the Andes regions in Bolivia, Chile and Peru (Stanschewski et al., 2021). Quinoa is a self-pollinated plant, but some cultivars may show cross-pollination of about 4-20% (Anchico-Jojoa et al., 2023). Quinoa can grow in hard and stressful conditions such as salinity and drought stresses (Anchico et al., 2020; Manjarres-Hernández et al., 2021). The grain yield of quinoa cultivars varies between 2.2-9.8 tons per hectare (Vásquez et al., 2024). Different cultivars have different stem diameter, plant height, and grain length and diameter (Manjarres-Hernández et al., 2021). The growth and development of quinoa is strongly influenced by day length and photoperiod, so that its growth period in different cultivars and environmental conditions is approximately 90-240 days (Apaza et al., 2015; González et al., 2015).

The main product of quinoa is its grain, which is called vegetable caviar because of its high nutritional value (Ng and Wang, 2021). Quinoa grains have a lower sodium content but higher levels of calcium, magnesium, potassium, iron, manganese, and zinc compared to common cereals such as wheat, barley, and maize (Singh, 2019; Ayaşan, 2020; Thiam et al., 2021; Abdelshafy et al., 2024). Quinoa contains 9.1-15.7 grams of protein, 4-7.6 grams of fat, and 8.8-14.1 grams of fiber per 100 grams of fresh weight (Nowak et al., 2016). In addition to quinoa grains, young quinoa leaves can be used as a fresh and cooked vegetable (Pathan et al., 2019). Therefore, due to the nutritional value and high production potential of quinoa, attention to this crop plant has increased worldwide to sustainably replace the nutrition of the growing world population (Gómez et al., 2021; Mohamed Ahmed et al., 2021).

Climate changes in the world along with drying and salinization of the soils have caused many problems such as the loss of large parts of suitable agricultural soils and, finally, the migration of farmers and villagers to the cities. Quinoa is a very valuable crop plant in terms of tolerance to many environmental stresses, including drought and salinity. It can be cultivated in hard conditions and may be able to solve a significant part of these problems. Since different quinoa varieties have a very different range of tolerance, from sensitive to resistance to environmental stresses, it is necessary to investigate their adaptability and stability under different environmental conditions and to identify and introduce compatible and stable varieties to each environment (Ruiz et al., 2014; Bazile et al., 2015).

Genotype × environment interaction is of particular importance for plant breeding researchers and one of the complex issues of breeding programs to identify high-yielding and stable genotypes (Dessie et al., 2020; Teressa et al., 2021). Evaluation of genotype × environment interaction is necessary to introduce genotypes with higher average grain yield and lower fluctuations (stable) in different environments (Tariku et al., 2013; Bocianowski et al., 2019). Powerful methods are needed to investigate genotype × environment interaction and determine the stability of genotypes. There are many different methods, including univariate and multivariate methods, to evaluate the stability of genotypes, which one or more methods can be used according to the experimental conditions. Among all stability analysis methods, it seems that two multivariate methods, additive main effects and multiplicative interactions or AMMI (Crossa et al., 1991; Gauch, 2006), and genotype plus genotype by environment interaction or GGE-biplot (Yan, 2001; Yan et al., 2010) are better and more successful methods to identify high-yielding and stable genotypes.

Kempton (1984) was the first researcher to use the AMMI model to analyze grain yield data. The AMMI method is a combination of analysis of variance and principal components analysis, in which analysis of variance is used to determine the main effects of genotype and environment, and principal components analysis is used to determine interaction effects. This analysis is an effective method to investigate the stability of genotypes in different environments because it calculates a large part of the sum of squares of the genotype × environment interaction and separates the main and interaction effects (Gauch, 2006). Considering that the effect of the environment is very large in most cases and cannot be used, removing the effect of environment and focusing on the main and genotype × environment interaction effects can be important (Yan and Kang, 2002; Gauch, 2006). Among the multivariate stability methods, the GGE-biplot graphical method, which is based on principal component analysis, is also a very useful tool for evaluating the role of genotypes, environments, and their interaction. To evaluate the stability of genotypes in this method, the effects of genotype and genotype × environment interaction are used to obtain more reliable results (Yan and Kang, 2002; Yan et al., 2007). The presence of different graphs in this method allows a better interpretation of the results, so that, it is possible to identify genotypes with higher grain yield and general stability for all environments, genotypes with specific stability for each of the target environments, and the best environments using the GGE-biplot tool (Yan and Kang, 2002; Yan et al., 2007; da Silva et al., 2021).

Many researchers have used AMMI and GGE-biplot methods to investigate the adaptability and stability of cultivars and genotypes in quinoa (Ali et al., 2018; Thiam et al., 2021; Al-Naggar et al., 2022; Anchico-Jojoa et al., 2023), maize (Al-Naggar et al., 2021; Patel et al., 2023; Greveniotis et al., 2023a), wheat (Mohamed et al., 2022; Omrani et al., 2022; Gupta et al., 2023), rice (Hasan et al., 2022; Abebe et al., 2023) and sorghum (Al-Naggar et al., 2018a, b). Ali et al. (2018) used univariate and multivariate methods to evaluate the stability of five quinoa genotypes in ten different environments in Egypt during two crop years, 2016 and 2017. They reported that the stability parameters and AMMI method were similar in identifying stable genotypes. Thiam et al. (2021) used the AMMI method to evaluate the stability of 14 quinoa genotypes in five different environments in Morocco, and classified the genotypes into three groups, stable, relatively stable and unstable. Al-Naggar et al. (2022) studied the stability and adaptability of 37 quinoa genotypes in 12 environments under different nitrogen fertilizer sources and level conditions in Egypt. They reported that the results obtained from both AMMI and GGE-biplot methods were different and each method introduced three different genotypes as stable and adaptable genotypes. Anchico-Jojoa et al. (2023) reported a significant difference between environments, genotypes, and genotype × environment interaction using the AMMI method to evaluate the stability and adaptability of eight quinoa genotypes in Brazil and Colombia during 2018 and 2019 and introduced four quinoa genotypes with high grain yield and general adaptability.

A few studies have been conducted in Iran on the adaptability and stability of quinoa. Bagheri et al. (2021) studied the stability and adaptability of ten different quinoa genotypes in four environments in cold and temperate locations of Iran during the 2017 and 2018 crop years, and introduced the stable and adaptable genotypes using the AMMI method. Also, Etaati et al. (2023) evaluated the adaptability and stability of ten quinoa genotypes in different locations of Iran using different parametric and non-parametric methods, and identified a high-yielding, stable and adaptable genotype.

Iran is a vast country located in the Asian continent and in the Middle East region and has a very diverse climate but with an average annual temperature of 17.6°C and an average rainfall of 266 mm (Abbasi et al., 2019), it is generally classified as a hot and dry climate. Therefore, it will be successful to cultivate plants that can tolerate the hard conditions caused by drought and heat stresses in most regions of Iran. It seems that the cultivation of quinoa as a valuable crop, especially in terms of tolerance to environmental stresses such as drought, can be successful in these conditions. Quinoa is a new crop plant in Iran, and suitable genotypes for different locations have not been introduced. The objective of the current study was to identify high-yielding and stable genotypes for the study regions, as well as to compare multivariate AMMI and GGE-biplot methods and introduce the best method for identifying stable and high-yielding genotypes.

## 2 Materials and methods

### 2.1 Plant materials and experimental locations

The plant materials of this study were 40 quinoa genotypes originating from Peru, Chile and Bolivia (**Table 1**). All genotypes were obtained from the IPK Gene Bank, Leibniz Institute of Plant Genetics and Crop Plant Research, Germany. The experiment was carried out in a randomized complete block design (RCBD) with three replications in four locations (Buin Zahra and Takestan in Qazvin province, and Kuhdasht and Poldokhtar in Lorestan province, Iran), during 2022-2023 and 2023-2024 cropping years. The geographical and climatic characteristics of the experimental locations are presented in **Table 2**.

**Table 1:** Quinoa genotypes studied in this research.

Row	Genotype	ID (Code)	Origin	Seed color	Altitude (m)	Row	Genotype	ID (Code)	Origin	Seed color	Altitude (m)
1	CHEN67	D2190	Peru	Brown	3000	21	CHEN167	D9346	Chile	Yellow	2600
2	CHEN68	D2191	Peru	Golden-brown	3030	22	CHEN171	D9350	Chile	Bright-white	2500
3	CHEN71	D2196	Chile	Light brown	2500	23	CHEN172	D9351	Peru	White	3000
4	CHEN83	D2194	Bolivia	Bright-white	3800	24	CHEN179	D9358	Chile	White	2700
5	CHEN84	D2195	Bolivia	White	3870	25	CHEN182	D9392	Peru	White	3200
6	CHEN89	D5078	Bolivia	Bright	3700	26	CHEN205	D9416	Chile	White	2600
7	CHEN90	D5079	Chile	White	2800	27	CHEN206	D9417	Chile	Golden	2800
8	CHEN91	D5081	Bolivia	Golden	3800	28	CHEN207	D9418	Chile	Bright	2700
9	CHEN115	D9316	Bolivia	White	3870	29	CHEN209	D9420	Chile	Bright-white	2900
10	CHEN119	D9319	Bolivia	Whitish-yellow	3800	30	CHEN210	D9421	Chile	White	2900
11	CHEN121	D9336	Chile	Yellow	2900	31	CHEN212	D9426	Chile	Golden	3000
12	CHEN123	D9428	Peru	White	3200	32	CHEN214	D9429	Peru	White	3100
13	CHEN126	D9339	Peru	Bright	3150	33	CHEN215	D9730	Peru	Bright	2800
14	CHEN128	D9320	Chile	Whitish-yellow	2600	34	CHEN216	D9431	Peru	White	3150
15	CHEN133	D9361	Bolivia	Yellow	3850	35	CHEN217	D9432	Chile	Bright	2500
16	CHEN146	D9374	Bolivia	Bright-white	3860	36	CHEN218	D9434	Chile	Whitish-yellow	2600
17	CHEN151	D9382	Chile	White	3000	37	CHEN220	D9439	Peru	Yellow	3000
18	CHEN154	D9385	Peru	White	3100	38	CHEN223	D9442	Chile	Bright-white	2700
19	CHEN156	D9390	Chile	Golden	2700	39	CHEN225	D9443	Peru	White	3200
20	CHEN159	D9376	Bolivia	Brown	3770	40	CHEN255	D9502	Chile	White	2900

**Table 2:** Geographical location, elevation and soil chemical analysis for the experimental plots (Source: I.R. of Iran Meteorological Organization (https://ndc.irimo.ir/far/wd/4641%D8%A8%D8%A7%D8%B1%D8%B4.html).

Location	Year	Spring temperature (°C)			Summer temperature (°C)			Solar Radiation (h)		Rainfall (mm)	Longitude	Latitude	Elevations (m)
		Min	Max	Average	Min	Max	Average	Spring	Summer				
Buin Zahra	2022	10.1	25.0	17.5	17.3	34.5	25.9	779.6	1051.4	241.9	50^°^4^´^E	35^°^46^´^N	1210
	2023	11.9	26.1	19.0	17.3	34.5	25.9	900.4	1084.5	224.4	
Takestan	2022	9.2	23.3	16.3	16.5	32.6	24.5	761.1	1081.2	269.2	46^°^42^´^E	36^°^4^´^N	1265
	2023	11.0	24.4	17.7	16.4	32.2	24.3	866.2	1034.0	272.8	
Kuhdasht	2022	9.6	29.0	19.3	17.8	35.2	26.5	797.2	1091.3	454.6	47^°^39^´^E	33^°^31^´^N	1197
	2023	9.9	29.3	19.6	18.2	36.4	27.3	834.3	1105.3	405.4	
Poldokhtar	2022	19.9	33.6	26.8	24.1	38.3	31.2	847.4	1149.7	368.7	47^°^43^´^E	33^°^9^´^N	714
	2023	18.3	32.9	25.6	21.2	38.6	29.9	934.5	1203.1	443.5	

To provide suitable moisture for plowing as well as to stimulate the germination and emergence of weed seeds buried in the soil for better control of weeds, the experimental field was irrigated twice before tillage. After irrigation and reaching soil moisture to the field capacity, field preparation including plowing, discing, and leveling was performed. The physical and chemical characteristics of the experimental soil are presented in **Table 3**. Before planting and at the same time of plowing, 50, 100 and 65 kg.ha^-1^ of N, P and K were used from the CH_4_N_2_O, P_2_O_5_ and K_2_O sources, respectively. Moreover, 50 kg.ha^−1^ of urea fertilizer was used as a topdress at two stages: half in the 6-8 leaf stage and the other half before the flowering stage. The seeds of all genotypes in all four locations and in both years, were planted on April 10. The length and width of the experimental plots were 5 and 3 m and the distance between the rows and between plants on the rows were 50 and 25 cm, respectively (Bazile et al., 2016). A sprinkler irrigation system was used to irrigate quinoa plants. The first irrigation was done after sowing the genotypes, the second and third irrigation were set 4 days apart, while subsequent irrigation was scheduled between 7 and 15 days later. Soil moisture was monitored using tensiometers, and irrigation was applied when moisture levels fell below 60-80% of field capacity. The irrigation schedule was adjusted based on seasonal rainfall and key growth stages, such as germination, flowering, and seed filling, to enhance crop resilience and maximize yield. Moreover, weeds were manually removed for the entire growing season to keep the soil bare. To control pests, especially the Caradina armyworm (*Spodoptera exigua*), two liters per hectare of Cypermethrin 40% insecticide were used in two stages before the flowering phase. Harvesting was done at the full maturity of the grains. To measure the grain yield, all plants of each plot after removing the border effect were harvested, and the weight of grains was calculated in kg.ha^-1^.

**Table 3:** Soil characteristics of the experimental fields.

Location	Clay (%)	Silt (%)	Sand (%)	Available K (ppm)	N (%)	Available P (ppm)	Organic carbon (%)	pH	EC (ds/m)
Buin Zahra	16	22	62	273	0.2	4.2	1.6	7.9	6.8
Takestan	22	26	52	510	0.5	11.6	4.7	6.2	7.4
Kuhdasht	39	40	21	325	0.1	4.6	0.87	7.5	4.6
Poldokhtar	38	35	27	123	0.3	5.2	0.45	7.6	3.6

### 2.2 Data analysis

AMMI and GGE-biplot methods were used to investigate the stability of the grain yield of genotypes. In the AMMI method, the main effects of genotypes (G) and environments (E) as well as G×E interaction were separated based on the following statistical model (Equation 1) as described by Gauch (1988):


(1) $${\text{Y}}_{ij}=\text{µ}+{\text{g}}_{i}+{\text{e}}_{j}+\sum_{\text{n}=1}^{\text{p}}{\text{λ}}_{n}{\text{δ}}_{in}{\text{η}}_{jn}+{\text{θ}}_{ij}+{\text{ϵ}}_{ij}$$

Where Y_ij_ is the grain yield of the i^th^ genotype in the j^th^ environment, µ is the total mean, g_i_ is the main effect of the genotype, e_j_ is the main effect of the environment, Λ_n_ is the singular value of the n^th^ principal component, δ_in_ is the eigen vector score for the i^th^ genotype of the n^th^ principal component of the G×E interaction, ɳ_jn_ is the eigenvector score for the j^th^ environment of the n^th^ principal component of the G×E interaction, Θ_ij_ is the residual effect and ε_ij_ is the experimental error.

The F-test is used to check the significance of the sources of variation, assuming normality and independence of the linear model. Because the AMMI model is a reduction model and the eigen values do not have the chi-square distribution, it is necessary to use corrected F-tests. Therefore, the significance tests of G×E interaction components were performed using the FR or Cornelius test based on the Equation 2 (Cornelius et al., 1992):


(2) $${\text{F}}_{R}=\frac{{\text{SS}}_{GEI}-\sum_{k=1}^{n}{\hat{\lambda }}^{2}}{{\text{fs}}^{2}}$$

Where SS_GEI_ is the sum of squares of the G×E interaction, $\sum_{k=1}^{n}{\hat{\lambda }}^{2}$ is the sum of squares of the nth principal component, f is the Cornelius degree of freedom (Equation 3), and S2 is the error mean square.


(3) f=(g-1-m)(e-1-m)

In this formula, g is the number of genotypes, e is the number of environment and m is the number of main components. FR or Cornelius test was performed based on IML procedure in SAS software (SAS Institute, 2017).

AMMI stability value (ASV) was calculated using the Equation 4 (Purchase et al., 2000):


(4) $${\text{ASV}}_{i}=\sqrt{\left[\frac{{\text{SS}}_{IPCA1}}{{\text{SS}}_{IPCA2}}{\text{(IPCA}1\text{ Score)}}^{2}+{\text{(IPCA}1\text{ Score})}^{2}\right]}$$

Where IPCA_1_ and IPCA_2_ scores are the values of the first and second interaction principal components for each genotype, and SS_IPCA1_ and SS_IPCA2_ are the sum of squares of the first and second interaction principal components, respectively.

In the AMMI method, a distribution diagram of genotypes and environments is drawn based on the average grain yield and the first principal component score (AMMI1 biplot) to identify stable and high-yielding genotypes. Additionally, a diagram resulting from the scores of the first two principal components is drawn to identify stable genotypes in all environments and to identify unstable genotypes (Gauch, 1988).

The GGE-biplot method, which is actually a type of principal component analysis for the sum of the main effect of genotype and the interaction effect of G×E, and uses the singular value decomposition (SVD), was performed based on the following statistical model (Yan, 2001):


(5) $${\text{Y}}_{ij}=\text{µ}+{\text{e}}_{j}+{\text{λ}}_{1}{\text{δ}}_{i1}{\text{η}}_{1j}+{\text{λ}}_{2}{\text{δ}}_{i2}\eta_{2j}+\epsilon_{\text{ij}}$$

In Equation 5, Y_ij_ is the grain yield of the i^th^ genotype in the j^th^ environment, µ is the total mean, e_j_ is the main effect of environment, Λ_1_ and Λ_2_ are the singular values of the first two principal components, PC1 and PC2, respectively, δ_i1_ and δ_i2_ are the eigen vectors of the i^th^ genotype for PC1 and PC2, respectively, ɳ_1j_ and ɳ_2j_ are the eigen vectors of the j^th^ environment for PC1 and PC2, respectively, and ε_ij_ is the residual value that cannot be explained by G×E interaction effect.

In the GGE-biplot method, the Which-Won-Where diagram was drawn to identify mega-environments and stable genotypes in each mega-environment. The GGE-biplot vector view was drawn to study the relationships between the studied environments. The GGE-biplot mean versus stability diagram was drawn to compare the studied genotypes with ideal genotypes, and the environment ranking pattern was drawn to compare the studied environments with the ideal environment. Using the graphs obtained from the GGE-biplot, the best environment, the best genotype for each environment, and the general stable genotype with higher grain yield for all environments were identified (da Silva et al., 2021). Stability analysis based on both AMMI and GGE-biplot methods was performed using PB Tools version 1.4 software (http://bbi.irri.org/products).

## 3 Results

### 3.1 AMMI analysis

**Figure 1:**
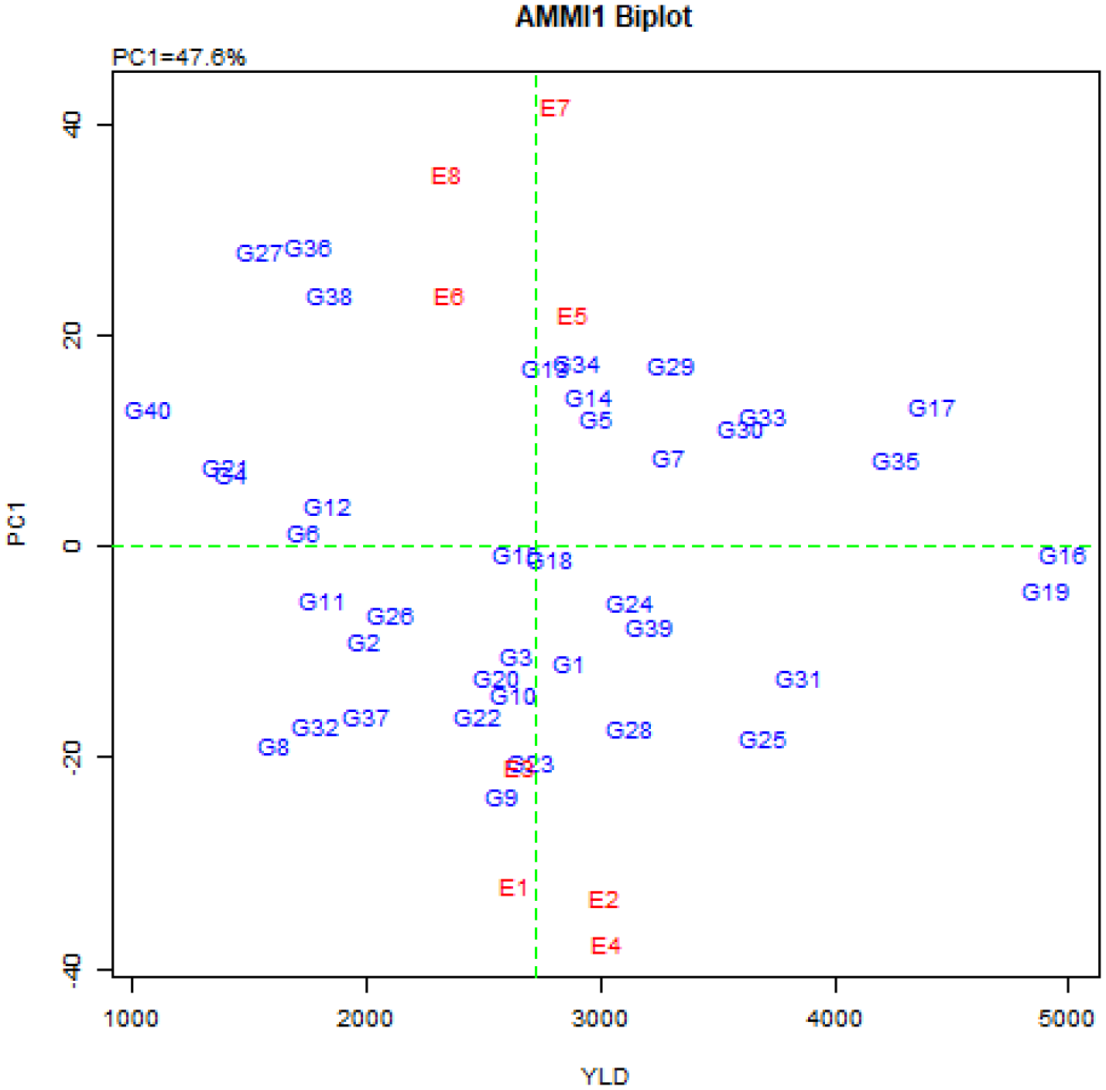
AMMI1 biplot of quinoa genotypes and environments based on the first principal component and grain yield.

The combined variance analysis of grain yield data of 40 quinoa genotypes in eight environments (**Table 4**) showed that the effects of G, E, and G×E interaction were highly significant (P ≤ 0.01), indicating a significant difference in average grain yield among environments and genotypes, as well as the fluctuation of the grain yield of quinoa genotypes from one environment to another. Therefore, it is possible to identify high-yielding genotypes with general stability for all environments as well as suitable and high-yielding genotypes for each environment using stability analysis. The results of the AMMI analysis of variance (**Table 4**) also showed that G, E, and G×E interaction explained 63.0%, 4.3% and 29.7% of the total variation, respectively. The higher contribution of the genotype can be attributed to the high genetic diversity of the studied genotypes. Also, the description of 29.7% of the total variation by G×E interaction can be related to the difference between genotypes and the difference in climatic conditions of the studied environments, so that these differences led to different reactions of quinoa genotypes in different environments. Separation of the G×E interaction based on the principal component analysis method also showed that the effects of the first six principal components were significant and explained 47.6%, 22.5%, 9%, 7%, 6%, and 4.3% of the G×E interaction variance, respectively. Also, these six principal components justified 14.1%, 6.7%, 2.7%, 2.1%, 1.8%, 1.3%, and 1.1% of the total variance, respectively (**Table 4**). The distribution diagram of genotypes and environments based on average grain yield and the first principal component score (AMMI1 biplot) is presented in **Figure 1**. Based on this biplot, by increasing the contribution of the first principal component in explaining the variance of the G×E interaction, stable genotypes can be identified more accurately.

**Table 4:** AMMI analysis of variance for grain yield of 40 quinoa genotypes across eight environments.

Source of variation	df	Sum of square	Mean square	df _Cornelius_	F _Cornelius_	Total variance proportion (%)	G×E variance proportion (%)
Treatment	319	1330815058	4171834^**^			97.1	–
Genotype	39	864054870	22155253^**^			63.0	–
Environment	7	59506151	8500879^**^			4.3	–
Block (Environment)	16	3342338	208896^**^			0.2	–
Interaction	273	407354037	1492139^**^			29.7	–
IPCA1	45	193814423	4306987	228	7.90^**^	14.1	47.6
IPCA2	43	91489321	2127659	185	5.56^**^	6.7	22.5
IPCA3	41	36669307	894373.3	144	5.00^**^	2.7	9.0
IPCA4	39	28449892	729484.4	105	4.57^**^	2.1	7.0
IPCA5	37	24611587	665178	68	4.01^**^	1.8	6.0
IPCA6	35	17420554	497730.1	33	3.81^**^	1.3	4.3
Residual	33	14898953	451483.4			1.1	3.7
Error	624	37002556	59298.97			2.7	–
Total	959	1371159952	1429781			–	–

The average grain yield of the studied environments along with the IPCA_e_1 and IPCA_e_2 scores and the AMMI stability values (ASVs) are presented in **Table 5**. The environments of Buin Zahra and Takestan in 2022 had the lowest ASV values. In other words, the yield fluctuations of genotypes in these two environments were less than the other environments, but these environments had a lower average grain yield than the total mean (2721 kg.ha^-1^). Among the studied environments, however in 2023, Takestan and Buin Zahra with 3030 and 3021 kg.ha^-1^, respectively, had a higher average grain yield than the total mean (**Table 5**; **Figure 1**). The comparison of IPCA_e_1 and IPCA_e_2 scores also showed that Takestan and Poldokhtar environments had the lowest and highest values for the first and second components in 2022, respectively, indicating that quinoa genotypes had the lower and higher fluctuations in these environments compared to the other environments, respectively (**Table 5**). ASV values also showed that Takestan was a better environment and quinoa genotypes had less fluctuations in this environment, while Poldokhtar was not a suitable climate for the studied genotypes (**Table 5**).

**Table 5:** Mean, IPCAe1and IPCAe2 scores, and AMMI stability value (ASV) of eight environments (four locations and two years).

Location	Year	ID (Code)	Mean (kg.ha^-1^)	IPCA_e_1	IPCA_e_2	ASV_e_
Buin Zahra	2022	E1	2641	-31.97	-3.82	46.69
	2023	E2	3021	-33.17	-24.17	53.98
Takestan	2022	E3	2663	-20.74	7.91	31.20
	2023	E4	3030	-37.54	4.46	54.80
Kuhdasht	2022	E5	2887	22.21	34.47	47.26
	2023	E6	2359	23.84	42.26	54.67
Poldokhtar	2022	E7	2814	41.93	-30.86	68.38
	2023	E8	2356	35.43	-30.25	59.78
Total mean	2022	–	2751	–	–	–
	2023	–	2692	–	–	–

**Figure 2:**
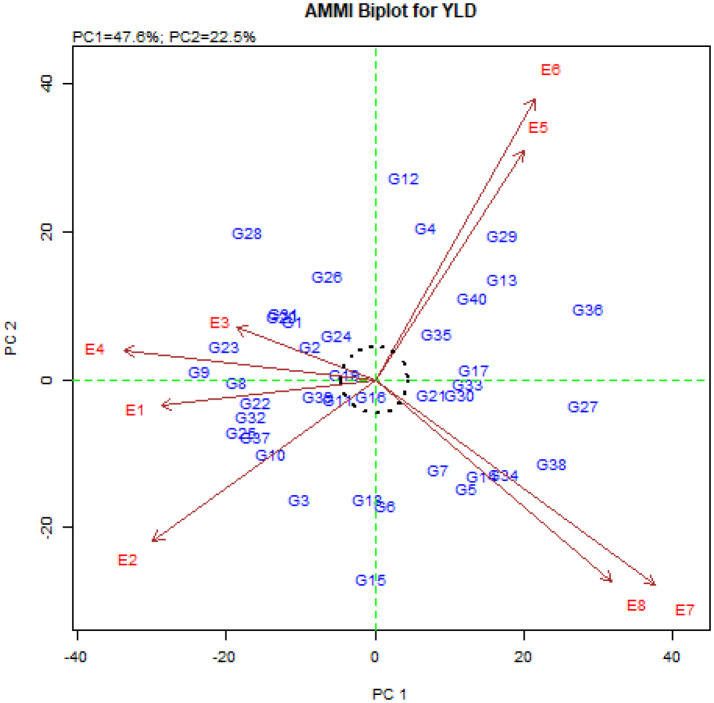
AMMI biplot based on the first and second principal components.

The average grain yield, IPCA_g_1 and IPCA_g_2 scores as well as the ASV values for the studied genotypes are shown in **Table 6**. The average grain yield of quinoa genotypes ranged from 1070 kg.ha^-1^ in the G40 genotype to 4977 kg.ha^-1^ in the G16 genotype (**Table 6**). Genotypes G15, G16, G18, G6, G12, G19, G11, G24, G26 and G4 had the lowest IPCA_g_1 score, respectively (**Table 6**). The ASV index also identified genotypes G16, G19, G11, G24, G21, G39, G2, G35, G18, and G30 with the lowest score as the stable genotypes (**Table 6**). It should be noted that the ASV index emphasizes only the stability aspect of the genotypes, while the grain yield of the genotypes should also be considered. Therefore, among the above-mentioned genotypes, genotypes G16, G19, G35, G30, G39, G24, and G18 (with higher grain yield than total average and located at the origin of the AMMI biplot) are introduced as general stable genotypes (**Figure 2**). In contrast, genotypes G36, G27, G38, G9, G28, G29, G23, G34, G13, and G12 with the highest ASV value (**Table 6**) and located in the farthest points from the origin of the biplot (**Figure 2**), are introduced as the most unstable genotypes in the studied environments.

**Table 6:** Mean, IPCA-1 and IPCA-2 scores, and AMMI stability value (ASV) of 40 quinoa genotypes for grain yield.

Genotype	Mean (kg.ha^-1^)	IPCA_g_1	IPCA_g_2	ASV_g_	Genotype	Mean (kg.ha^-1^)	IPCA_g_1	IPCA_g_2	ASV_g_
G1	2871	-10.99	8.01	17.89	G21	1404	7.60	-2.00	11.24
G2	1989	-8.79	4.67	13.61	G22	2476	-16.11	-2.92	23.62
G3	2646	-10.21	-16.15	21.94	G23	2706	-20.37	4.49	29.98
G4	1418	6.86	20.66	22.94	G24	3125	-5.17	6.15	9.72
G5	2978	12.35	-14.54	23.11	G25	3699	-18.02	-7.01	27.14
G6	1730	1.40	-16.92	17.04	G26	2101	-6.36	14.03	16.80
G7	3290	8.55	-12.13	17.38	G27	1547	28.00	-3.44	40.88
G8	1607	-18.68	-0.23	27.18	G28	3131	-17.22	20.09	32.11
G9	2582	-23.61	1.31	34.38	G29	3298	17.19	19.51	31.72
G10	2629	-14.00	-9.94	22.67	G30	3603	11.41	-1.93	16.71
G11	1816	-5.03	-2.50	7.73	G31	3854	-12.36	9.03	20.13
G12	1839	3.97	27.36	27.96	G32	1783	-16.80	-4.82	24.92
G13	2769	17.03	13.66	28.30	G33	3698	12.51	-0.45	18.21
G14	2953	14.29	-12.84	24.44	G34	2903	17.40	-12.83	28.39
G15	2636	-0.50	-26.89	26.90	G35	4263	8.35	6.35	13.72
G16	4977	-0.58	-2.11	2.27	G36	1752	28.63	9.70	42.77
G17	4418	13.32	1.49	19.45	G37	2001	-16.04	-7.63	24.55
G18	2785	-0.98	-16.18	16.24	G38	1842	23.92	-11.24	36.58
G19	4901	-4.11	0.81	6.03	G39	3206	-7.59	-2.23	11.26
G20	2556	-12.42	8.50	19.97	G40	1070	13.12	11.12	22.10

### 3.2 GGE-biplot analysis

**Figure 3:**
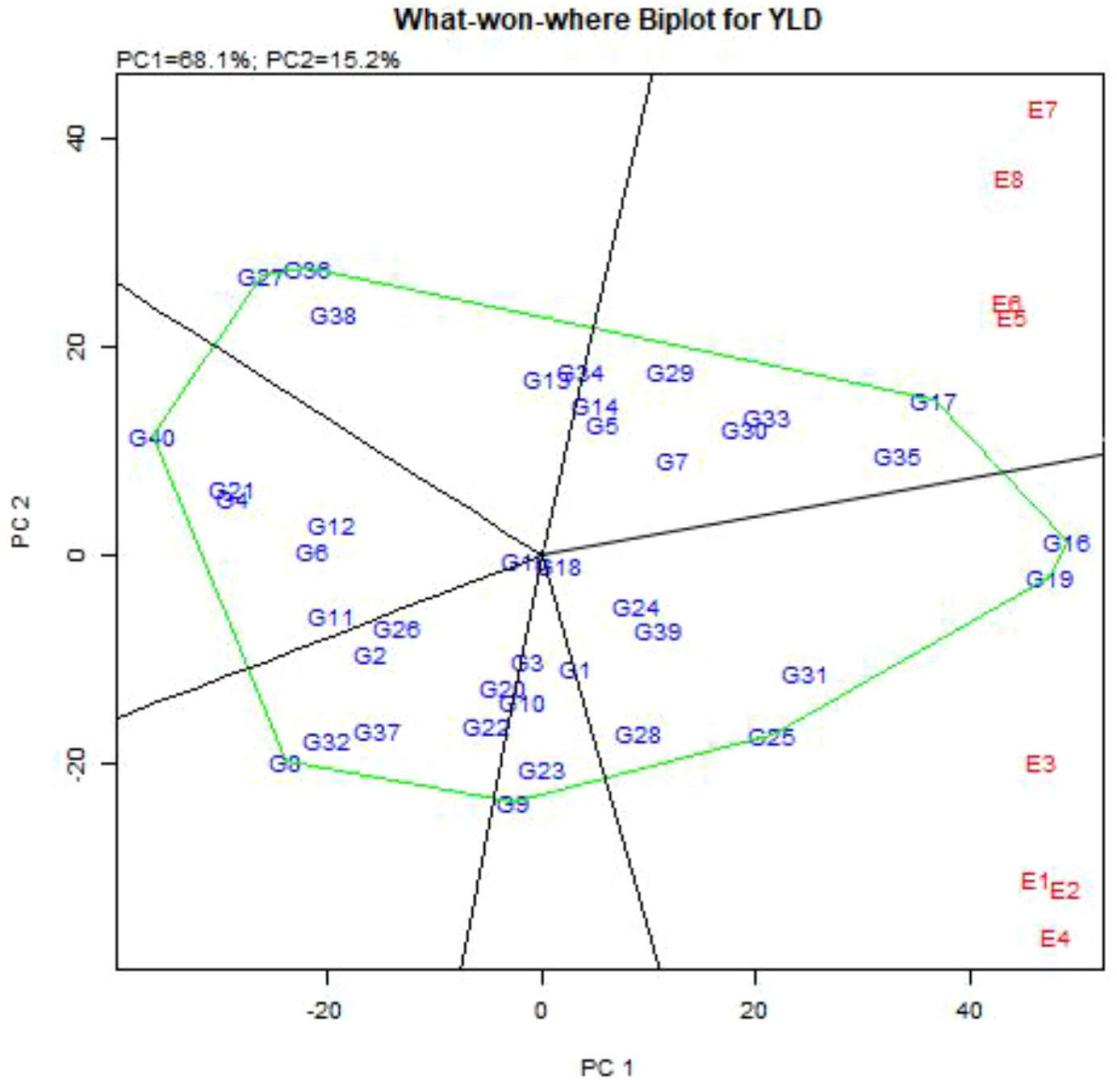
“Which-Won-Where” pattern of GGE-biplot polygon view to determine the superior genotypes in different environments.

GGE-biplot analysis showed that the first two principal components accounted for 83.3% of the variance of G + G×E interaction, of which 68.1% and 15.2% were explained by the first principal component (PC_1_), and the second principal component (PC_2_), respectively. To determine the best genotypes for each of the studied environments, the GGE-biplot polygon view (Which-Won-Where pattern) was presented (**Figure 3**). GGE-biplot identified two mega-environments, including Buin Zahra and Takestan locations in both years (E1, E2, E3 and E4), and Kuhdasht and Poldokhtar in both years (E5, E6, E7 and E8), respectively. Also, genotypes G16, G19, G25, G9, G8, G40, G27, and G17 were placed in the vertices of polygon, but genotypes G16, G19, and G25 (with grain yield of 4977, 4901 and 3699 kg.ha^-1^, respectively) in the first mega-environment and genotype G17 (4418 kg.ha^-1^) in the second mega-environment were identified as superior genotypes with higher adaptability. Moreover, genotypes G31, G39, G24, G28, and G18 in the first mega-environment and genotypes G35, G33, G30, G7, G29, G5, G14, and G34 in the second mega-environment showed a high correlation with the genotypes of the vertices of polygon, and with relatively suitable grain yield were compatible genotypes to these environments, the Genotypes G9, G8, G40, and G27 were also placed in the vertices of polygon, but they did not produce high grain yield in any of the studied environments (**Figure 3**).

**Figure 4:**
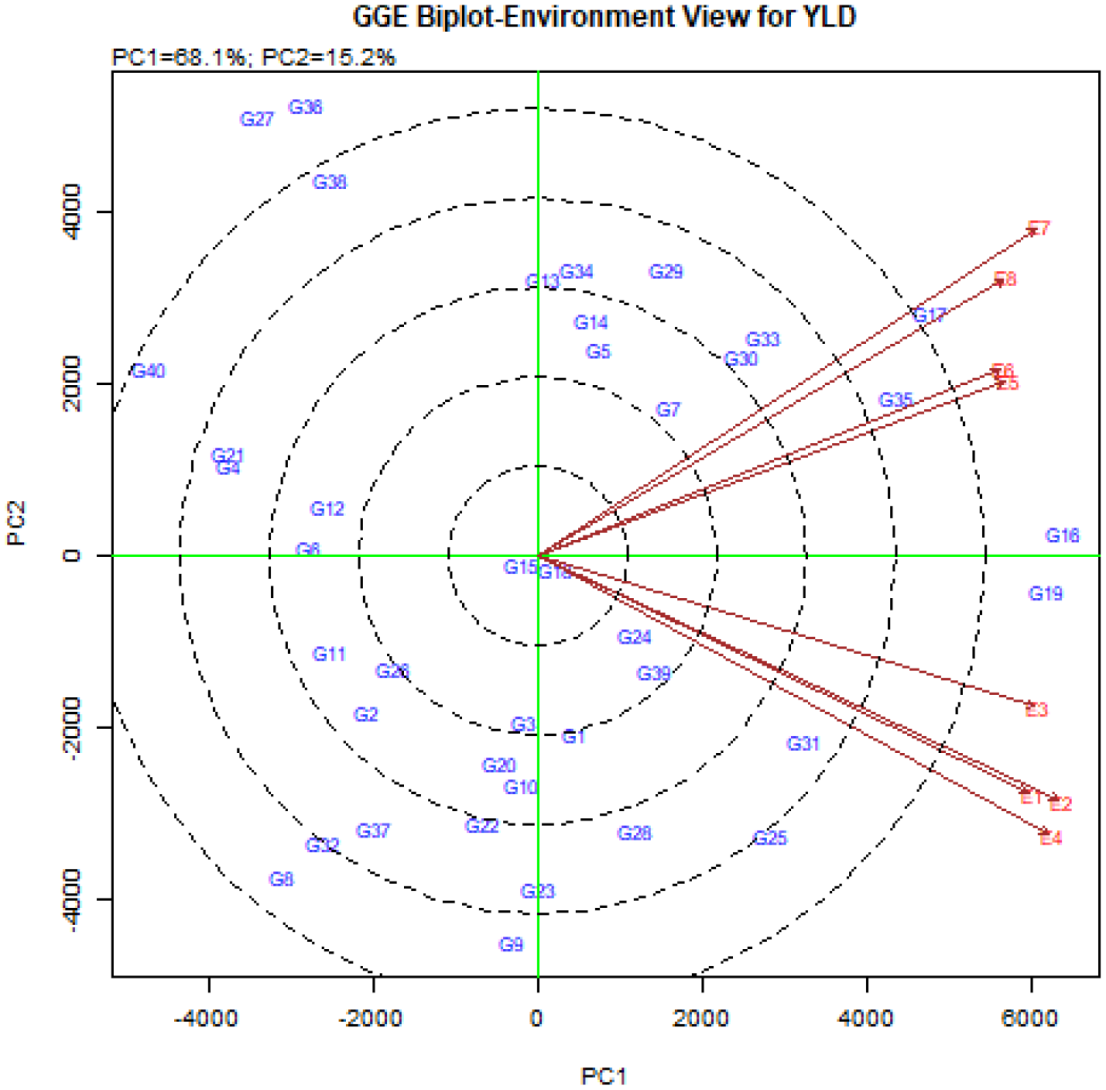
The vector view of GGE-biplot to study the relationships between the studied environments.

The vector view of GGE-biplot was used to study the relationships between environments (**Figure 4**). The results showed that the angle between the vectors of Buin Zahra and Takestan in both years (E1, E2, E3 and E4) as well as Kuhdasht and Poldokhtar in both years (E5, E6, E7 and E8) was small, which indicated a high correlation between them. The correlation between Buin Zahra and Takestan in both years with Kuhdasht and Poldokhtar in both years (E1, E2, E3 and E4 with E5, E6, E7 and E8) was low due to the large angle between their vectors. In total, the biplot study of the relationships between environments showed a high discrimination power in all the experimental environments. While all environments had long vectors, the vector length of Takestan and Buin Zahra in 2023 (E2 and E4) was longer than the vector length of other environments, indicating the high influence of these environments on differentiating genotypes compared to other studied environments. On the other hand, Kuhdasht had the smaller length vectors in both years (E5 and E6) compared to other environments (**Figure 4**), indicating lower fluctuations and higher stability of the grain yield of quinoa genotypes in this location.

**Figure 5:**
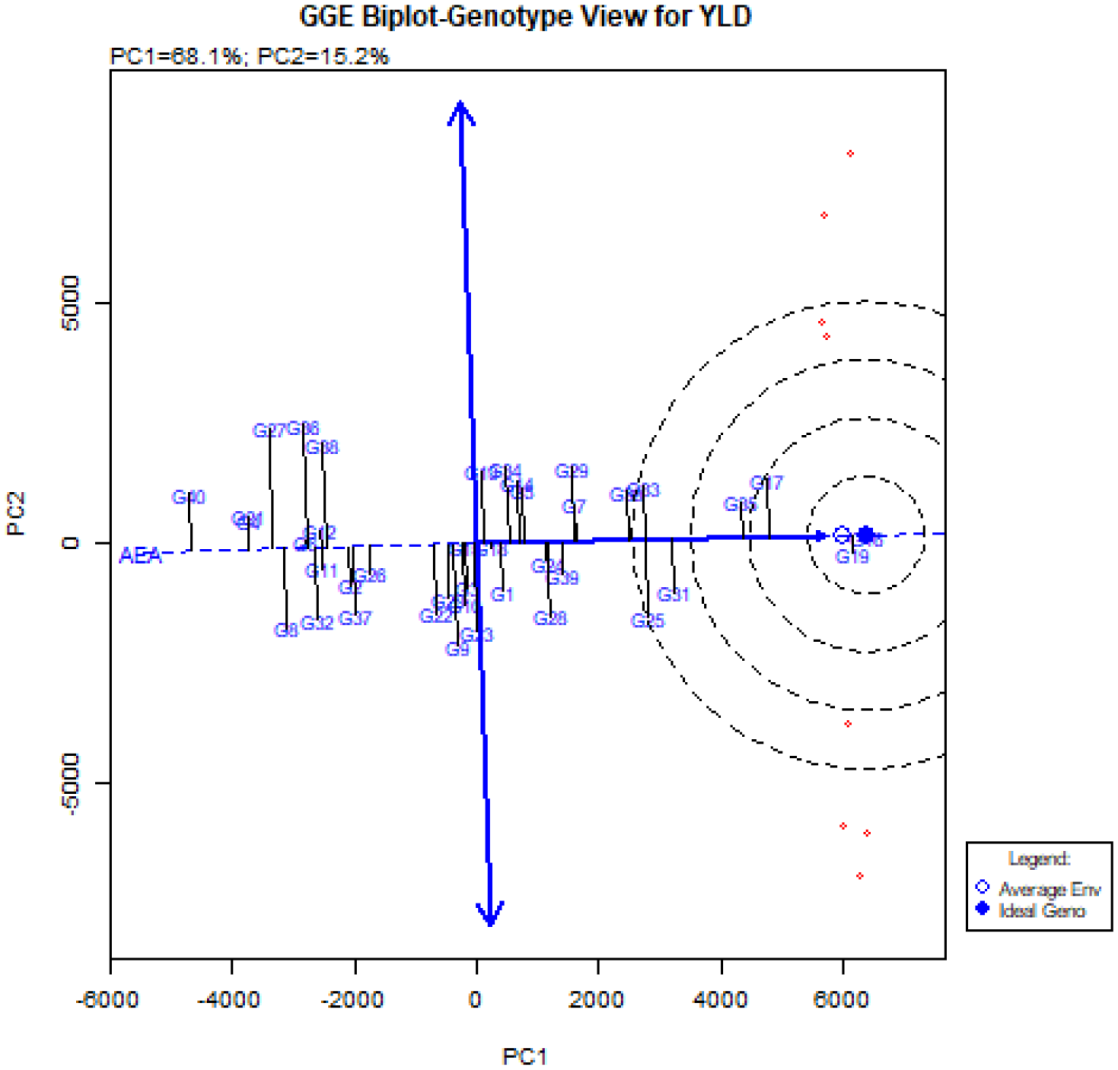
‘Mean vs. stability’ pattern of GGE-biplot to compare the studied genotypes with the ideal genotype.

The comparison of the ideal genotype with the studied quinoa genotypes is shown in **Figure 5**. The vertical line on the average environment axis (AEA) indicated with two arrows, is used to determine the stability of genotypes. Genotypes closer to the origin of AEA are more stable than genotypes closer to the end of this axis (Esan et al., 2023; Kebede et al., 2023). Therefore, genotypes G16, G19, G17, G35, and G31, which are closer to the AEA (**Figure 5**), were more stable than other genotypes in all environments. These genotypes with higher grain yield than total mean, had a relatively constant grain yield ranking in all environments. While genotypes G36, G27, G38, G9, G8, G32, G37, G28, G29, and G22 had less stability due to the longer distance from the AEA (**Figure 5**). Furthermore, the GGE-biplot genotype view showed that genotypes G16 and G19, followed by G17, G35 and G31, which were placed in the center of the concentric circles, were the most ideal genotypes in this experiment. In contrast, genotypes G40, G4, G21, G27, G36, G38, and G32 were the weakest quinoa genotypes in terms of grain yield and stability in this experiment.

**Figure 6:**
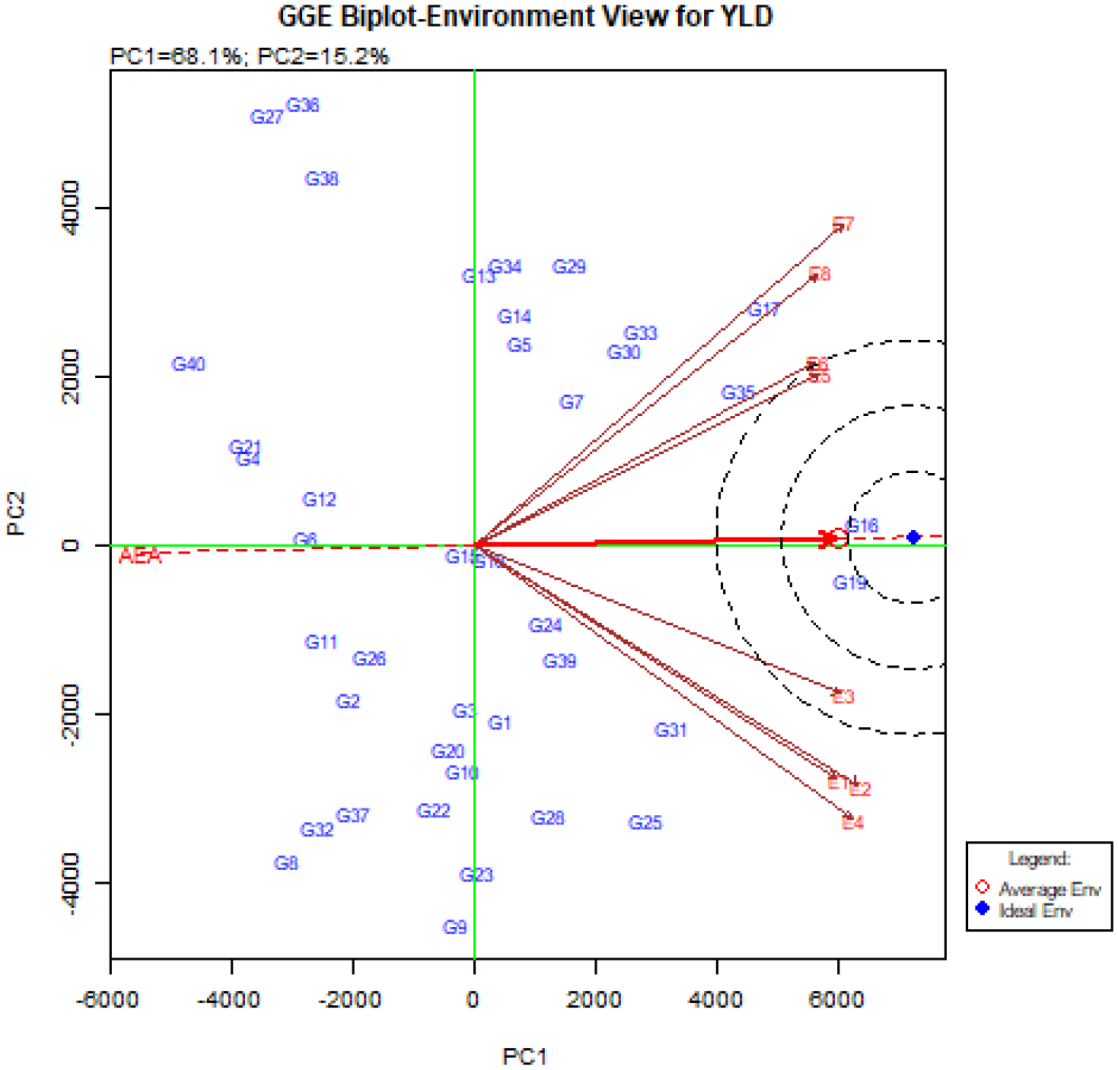
The GGE-biplot ‘environment ranking’ pattern to compare the studied environments with the ideal environment.

The GGE-biplot environment ranking pattern to compare the studied environments with the ideal environment is shown in **Figure 6**. The ideal environment (center of the concentric circles) as a representative of other studied environments, has the highest ability to differentiate genotypes (Esan et al., 2023; Kebede et al., 2023). The results showed that Takestan in 2022 (E3) can be introduced as the best environment due to the smallest distance from the ideal environment as well as the smallest angle with the AEA vector. After that, Kuhdasht in both 2022 and 2023 years (E5 and E6) were suitable environments for the studied quinoa genotypes in this experiment (**Figure 6**).

## 4 Discussion

Quinoa is known as a key crop for food security in many countries of the world, which can produce products in more than 120 countries, but on the one hand, the high climatic diversity in different regions and, on the other hand, the existence of environment × genotype interaction, requires the introduction and cultivation of stable and adapted genotypes to these areas (Alandia et al., 2020). There are various statistical methods to study genotype × environment interaction and identify stable genotypes, among which AMMI and GGE-biplot provide better and more interpretable results (Gauch et al., 2008). In the AMMI method, the genotype × environment interaction is separated from the environment and genotype effects, and then this interaction is analyzed using PCA (Zobel et al., 1988), While in the GGE-biplot method, only the environment effect is removed, and the total effects of genotype and genotype × environment interaction are used to identify stable genotypes, and by presenting different biplot, ideal and stable environments and genotypes are introduced (Yan et al., 2007).

The variance analysis of the data in this study showed that the effects of genotype, environment, and genotype × environment interaction were significant. The significant genotype × environment interaction indicates the fluctuation of the grain yield of the genotypes from one environment to another (Thiam et al., 2021; Allaoui et al., 2023; Ruswandi et al., 2023). This means that the grain yield of the genotypes was affected by environmental conditions and produced different grain yields in different environments. The separation of the contribution of each factor also showed that genotype, environment, and genotype × environment interaction justified 63.0%, 4.3%, and 29.7% of the total variation, respectively. Describing 30% of the total grain yield variation by genotype × environment interaction makes the necessity of stability analysis and identification of stable genotypes unavoidable. The results of this study were consistent with the results of many researchers who reported the contribution of the effects of genotype and genotype × environment interaction more than the environment effect (Hmwe et al., 2018; Katsenios et al., 2021; Hossain et al., 2023; Wodebo et al., 2023). On the other hand, some researchers also estimated the environmental effect is more than the effects of genotype and genotype × environment interaction (Ali et al., 2018; Wardofa et al., 2019; Thiam et al., 2021; Al-Naggar et al., 2022).

The description of nearly 50% of the variance of the genotype × environment interaction by the first component and nearly 71% of this variance by the first two components indicates the existence of a strong interaction between the studied genotypes and environments. Tekdal and Kendal (2018) also investigated the stability of 122 different durum wheat genotypes in two environments using the AMMI model, and they reported that genotype, environment, and genotype × environment interaction explained 59.8%, 3.5%, and 36.7% of the data variation, respectively. Other researchers also reported a high percentage of the first and second principal components in the AMMI method (El-Sadek, 2017; Ali et al., 2018; Allaoui et al., 2023; Hossain et al., 2023). The studied environments were located far from the origin of the AMMI biplot, which indicated strong interaction forces with the genotype, and the angles between the studied environments (**Figure 2**) indicate the distinctiveness in the selection of genotypes (Balakrishnan et al., 2016; Jain et al., 2019). Therefore, using the values of the first principal component, the mean grain yield of the genotypes (**Figure 1**), and the values of the first and second principal components (**Figure 2**), the AMMI diagram was drawn to show the effects of genotypes and environments and the distribution of genotypes in the eight studied environments. The results showed that the genotypes located on the right side of the AMMI1 diagram (**Figure 1**) had higher grain yield than the total mean. Among these genotypes, genotypes G16, G19, G17, and G35 produced the highest grain yield with 4977, 4901, 4418 and 4263 kg.ha^-1^, respectively. According to **Figure 2**, genotypes G25, G28, G31, G9, and G23 with Buin Zahra and Takestan environments in 2022 and 2023 (E1, E2, E3, and E4) and genotypes G34, G12, G14, and G5 with Kuhdasht and Poldokhtar environments in 2022 and 2023 (E5, E6, E7 and E8) were adaptable. Thiam et al. (2021) also identified the genotypes adapted to each environment using the graph obtained from the first two components in the AMMI model according to the proximity of the genotype to the environment vector. The difference in grain yield between Lorestan province (Kuhdasht and Poldokhtar) and Qazvin province (Takestan and Buin Zahra) can be attributed to several factors. Sparrow (*Passer domesticus*) infestation during the flowering and harvesting of quinoa had a greater impact on yield in Lorestan province due to the absence of fields or cover crops to deter sparrow attacks, while in Qazvin province, the cultivation of sunflowers alongside quinoa helped reduce the impact of sparrows. Additionally, the soil in Qazvin province was more suitable for quinoa cultivation, with a lighter texture compared to Lorestan province. Furthermore, high temperatures during flowering and physiological maturity in Lorestan province led to a decrease in the number of seeds and ultimately a decrease in grain yield.

The IPCA_g_ index is one of the parameters that is calculated to determine the degree of interaction of each genotype with the environment and to identify stable genotypes in the AMMI method. Based on this, genotypes with a large IPCA_g_1 Score (positive or negative) have a high interaction with the environment, and on the contrary, genotypes with an IPCA_g_1 score close to zero have a low interaction and are stable genotypes (Purchase et al., 2000). Investigation of the IPCA_g_1 values showed that genotypes G15, G16, G18, G6, G12, G19, G11, G24, G26, and G4 were the most stable studied genotypes in this experiment with the lowest IPCA_g_1 score, respectively. The AMMI stability value (ASV) is another parameter of the AMMI method that expresses the variation between genotypes so that genotypes with less ASV and close to zero are considered stable (Mohammadi and Amri, 2008; Kebede and Getahun, 2017). The ASV index, like the IPCA_g_1 index, identifies similar and identical stable genotypes. Considering that genotypes must be introduced that have high grain yield in addition to stability and fewer yield fluctuations in different environments, the examination of the grain yield of these genotypes showed that only genotypes G16, G19, G35, G30, G39, G24, and G18 had higher grain yield than the population means. These genotypes were also placed at the origin of the AMMI biplot (**Figure 2**) and as a result, stable genotypes with high general adaptability were introduced.

The results of the GGE-biplot method also showed that the first two principal components, explained 68.1% and 15.2%, respectively, and in total about 83% of the variation of the genotype and genotype × environment interaction. According to many researchers, the GGE-biplot method is one of the most appropriate methods to investigate the stability of genotypes (Chandrashekhar et al., 2020; Haile and Kebede, 2021; Greveniotis et al., 2023b) because in this method, by removing the environment effect as an uncontrollable source, the sum of the effects of genotype and the interaction of genotype × environment are used, and as a result, the total controllable effects are used to study the response of genotypes and identify stable genotypes. Many researchers have reported the high contribution of the first principal component in explaining diversity of the genotype and genotype × environment interaction in different plants, including quinoa (Afiah et al., 2018; Thiam et al., 2021; Hafeez et al., 2022), rice (Hasan et al., 2022; Ghazy et al., 2023), maize (Shojaei et al., 2022; Ninou et al., 2023), wheat (Elfanah et al., 2023; Han et al., 2024), barley (Akan et al., 2023) and sorghum (Wang et al., 2023) which was consistent with the results of this study.

Considering that about 83% of the variance of the genotype and genotype × environment interaction in this study were described by the first and second components, therefore, different biplot diagrams can be drawn to study the distribution of genotypes in different environments and to choose the best genotypes and environments. One of these diagrams is the polygon biplot which is presented in **Figure 3**, and it can be used to identify superior genotypes adapted to any environment (Gao et al., 2022; Esan et al., 2023). In this diagram, vertical lines are drawn from the origin of the biplot to each side of the polygon, and the studied genotypes and environments are divided into several sections so that the genotypes placed in each part of the polygon diagram have high specific adaptability with the environment of that part, especially the genotypes located at the vertices of the polygon, which have the highest specific adaptability with the respective environment (Bojtor et al., 2021; Sissoko et al., 2024). Based on the study, it was found that Poldokhtar was the most unfavorable environment for grain yield, while Takestan was the most favorable. Several genotypes were identified as superior in each environment, with some performing well in one region but not in another. This information can be valuable for selecting appropriate genotypes for different environmental conditions to optimize grain yield. In addition to the stable and high-yielding genotypes introduced in all locations, genotypes G7, G30, G33, and G34 can be introduced as superior genotypes in the Poldokhtar environment, which did not provide optimal yield in the Takestan environment. Also, genotypes G25, G31, and G39 had optimal grain yield in the Takestan environment, but produced low grain yield in the Poldokhtar environment.

Another diagram that is drawn in the GGE-biplot method is the biplot diagram to investigate the relationships between the studied environments. The cosine of the angle between the environment vectors in this diagram shows the correlation between environments. A zero-degree angle means +1 correlation, a 90-degree angle means no correlation, an acute angle means positive correlation and an obtuse angle means negative correlation (Yan and Tinker, 2006). In addition, based on the length of the environment vectors in this diagram, the studied environments can also be examined in terms of the power of distinguishing genotypes, so that the environments with a longer vector length have more power and the ability to differentiate (Esan et al., 2023; Kebede et al., 2023). Correlation between the eight studied environments in this experiment showed that the angle between the vector of Buin Zahra and Takestan environments in both cropping years (E1, E2, E3, and E4) and Kuhdasht and Poldokhtar in both cropping years (E5, E6, E7, and E8) is smaller, which means that these environments are more similar to each other and there is a high correlation between them, while the angle between the environment vectors of Buin Zahra and Takestan with the vectors of Kuhdasht and Poldokhtar environments in both cropping years (E1, E2, E3, and E4 with E1, E2, E3, and E4) is larger and indicates the greater difference between these environments and less correlation between them. Considering that the information obtained from environments with high correlation is similar and identical, therefore, to increase the efficiency of the experiments, one of the environments with high correlation can be selected and future experiments can be performed in this environment and eliminate other environments and finally reduce the costs of performing experiments (Sharma et al., 2020; Karuniawan et al., 2021; Ruswandi et al., 2021; Taleghani et al., 2023). The investigation of the length vector of the environment also showed that all the eight studied environments in this experiment had large length vectors, which meant a high discrimination power in all the experiment environments, However, the vector length of Takestan and Buin Zahra environments in 2023 (E2 and E4) was more than the other six environments, which indicated the greater impact of these environments in differentiating genotypes compared to other studied environments (**Figure 4**). In other words, Takestan and Buin Zahra environments in 2023 (E2 and E4) had a greater contribution to the formation of the genotype × environment interaction than the other six environments. Therefore, the length of the environment vectors is a measure to determine the stability of environments and there is an inverse relationship between them, so that an environment with a smaller environment vector size is more stable (Esan et al., 2023; Kebede et al., 2023). Taking into account that the length of the vectors of the Kuhdasht environment in both cropping years (E5 and E6) was smaller than the vectors of other environments, these environments had higher stability and the grain yield fluctuation of quinoa genotypes in these environments was less than in other environments. This environment can be used for planning to select superior quinoa genotypes (Ansarifard et al., 2020).

Another diagram drawn in the GGE-biplot method is the Biplot diagram to compare genotypes with the ideal genotype. Based on this diagram, the genotypes are ranked and placed in concentric circles, and the genotypes placed in the center of these concentric circles are known as the ideal genotypes. In addition, the distance from the Average Environment Axis (AEA) is also an indicator to determine the stability of genotypes and has an inverse relationship with stability (Kebede et al., 2023; Ninou et al., 2023). The unique feature of the GGE-biplot is that it allows the comparison of genotypes with the ideal genotype. Examining the ‘Mean vs. stability’ pattern (**Figure 5**) showed that the genotypes G16, G19, G17, G35, and G31 were placed in the center of the concentric circles, and in contrast, the genotypes G40, G4, G21, G27, G36, G38, and G32 were located farther away from the concentric circles. Genotypes G16, G19 and G17 had the highest mean and genotypes G40, G4, and G21 had the lowest mean in the environment. According to this figure, genotypes G16, G19, and G17 were recognized as ideal genotypes in this study. Following this, genotypes G35 and, G31 were identified as superior and stable genotypes. Finally, using **Figure 6**., ideal environments were identified. In this diagram, the smaller the angle of the environment vector with the Average Environment Axis (AEA) and the intended environment placed in concentric circles, the more ideal that environment is (Ye et al., 2019). Therefore, the Takestan environment in 2022 (E3) and then the Kuhdasht environments in 2022 and 2023 (E5 and E6) were recognized as ideal environments for cultivating the studied quinoa genotypes. Al-Naggar et al. (2022) also introduced ideal genotypes and ideal fertilizer environments for quinoa cultivation by evaluating 37 quinoa genotypes using this biplot. These environments can be used to improve quinoa genotypes because they consistently produce the highest grain yield. GGE-biplot diagrams allow the evaluation of environments and genotypes based on the power of differentiation and representation, so this method is more favorable than the AMMI method (Aktaş, 2016).

## 5 Conclusion

In this study, genotype × environment interaction and grain yield stability were investigated in 40 quinoa genotypes using AMMI and GGE-biplot methods. The variance analysis of the data in this study showed that the effects of genotype, environment, and genotype × environment interaction were significant. The significant genotype × environment interaction indicates the fluctuation of grain yield of genotypes from one environment to another due to the variation in climatic and edaphic factors. The breakdown of the contribution of each factor reveals that genotype accounts for 63.0% of the total variation, environment for 4.3%, and genotype × environment interaction for 29.7%. The fact that genotype × environment interaction explains 30% of the total grain yield variation underscores the importance of conducting stability analysis and identifying stable genotypes. The results of AMMI analysis showed that the six principal components explained of the variance of genotype × environment interaction. The description of nearly 50% of the variance of the genotype × environment interaction by the first component and nearly 71% of this variance by the first two components indicates the existence of a strong interaction between the studied genotypes and environments. Among the genotypes studied, genotypes G16, G19, G17, and G35 had the highest grain yield with 4977, 4901, 4418, and 4263 kg/ha respectively. Also, various statistical analyses showed that Pol Dokhtar was the most unfavorable and Takestan was the most favorable environment for grain yield. Based on AMMI graphs, genotypes G25, G28, G31, G9, and G23 were adaptable to the environments of Buin Zahra and Takestan, and genotypes G34, G12, G14, and G5 were adaptable to the environments of Kohdasht and Pol Dokhtar. The results of the GGE-biplot method also showed that the first two principal components, explained about 83% of the variation of the genotype and genotype × environment interaction. The polygon diagram (**Figure 3**) separated the environments studied in this experiment into two mega-environments. The first mega-environment included the environments of Buin Zahra and Takestan, while the environments of Kuhdasht and Poldokhtar were placed in the second mega-environment. Among the studied genotypes, genotypes G16, G19, and G25 had the highest specific adaptability with the first mega-environment, and genotype G17 with the second mega-environment. The grain yield analysis of 40 studied quinoa genotypes in this experiment with both AMMI and GGE-biplot analysis methods identified genotypes G16 and G19 as stable and high-yielding genotypes. Also, both AMMI and GGE-biplot methods were beneficial in studying genotype × environment interaction and identifying stable and high-yielding genotypes. However, the GGE-biplot method, due to presenting different graphs, determining mega-environments, and identifying ideal genotypes, was a more useful tool for stability analysis. High-yielding and stable genotypes identified in this experiment can be introduced as suitable cultivars for the studied environments.

## Data Availability

The raw data supporting the conclusions of this article will be made available by the authors, without undue reservation.
